# Challenges in digital medicine applications in under-resourced settings

**DOI:** 10.1038/s41467-022-30728-3

**Published:** 2022-05-26

## Abstract

Digital medicine tools, including medical AI, have been advocated as potential game-changers to solve long-standing healthcare access and treatment inequality issues in low and middle income countries. As these applications are increasingly becoming a reality, we connect here with researchers with experience in planning and deployment of these tools in under-resourced settings.

## Teaser

Digital medicine tools, including medical AI, have been advocated as potential game-changers to solve long-standing healthcare access and treatment inequality issues in low and middle income countries. For example, automated tools can fill gaps in trained healthcare workforce availability and even augment equipment capabilities. As these applications are increasingly becoming a reality, we connect here with researchers with experience in planning and deployment of these tools in under-resourced settings, to understand what the current bottlenecks are from a practical point of view. The experts involved are Walter H. Curioso (a digital health expert and biomedical informatics consultant, Universidad Continental, University of Washington), Daniel S.W. Ting (an expert in deep learning for medical imaging and digital innovation, Singapore Health Service, Duke-NUS Medical School), Bram van Ginneken (an expert in machine learning for medical image analysis, Radboud University) and Martin C. Were (an expert in digital health and medical informatics, Vanderbilt University Medical Center).

**Figure:**
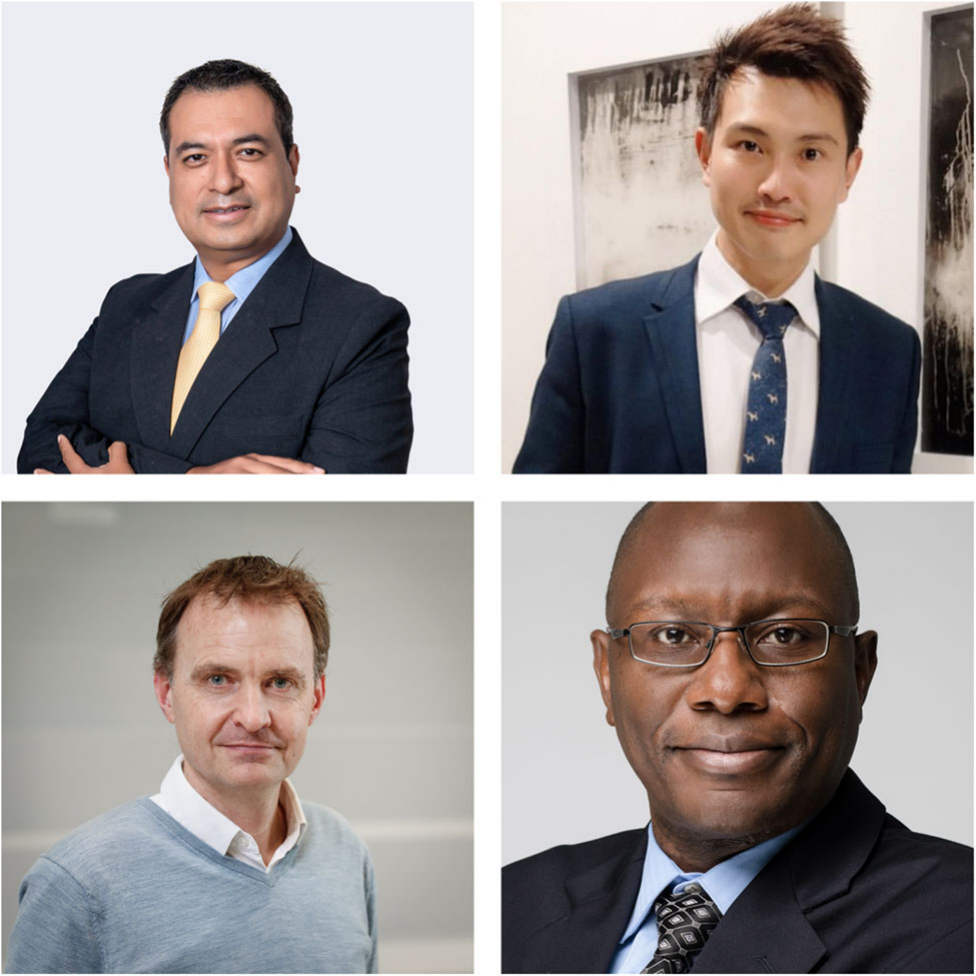
Walter H. Curioso, Daniel S.W. Ting, Bram van Ginneken, Martin C. Were

What are your main fields of expertize in the context of digital medicine? What project(s) have you been involved with in under-resourced settings?

**Walter H. Curioso:** My work has largely focused on the use of information and communication technologies to improve health care services in low-resource settings. I have experience in planning, developing, monitoring, and evaluating national public health projects related to digital health, including mobile health, electronic medical records, telemedicine, artificial intelligence, big data, and knowledge management. As a researcher on digital health, I provided advice to strategic institutions in digital health at the national and international level. I am currently part of the selected Roster of Experts on Digital Health appointed by the World Health Organization.

Regarding significant management experience in the public sector, I served as the General Director of the National Office of Statistics and Informatics at the Peruvian Ministry of Health, the highest position for a digital medicine professional at the government level in Peru. I proposed meaningful digital health policies and implemented multi-institutional national health initiatives related to electronic medical records, mobile health and telemedicine. I led a landmark program, the On-Line Birth Registration System, developed in conjunction with the Ministry of Health and the National Statistics Division. This system registers newborns in the delivery room and substantially simplifies the process required to obtain the birth certificate and further the National ID for the newborn. This standards-based system was implemented nationally both at private and public hospitals and is the main cornerstone of the health information system in Peru which helps obtain real-time statistics for timely decision making in the health sector. For example, a newborn with very low weight in a small health care center could be referred more efficiently to more complex hospitals with the aid of technologies. All the information captured at the small health care center automatically is gathered at the Ministry of Health for effective coordination and planning. This is just one example of how real-time data collection, and a simple technology can save lives, even in remote areas.

**Daniel S.W. Ting:** As the Director of the cluster AI program of the Singapore Health Service (SingHealth), my research focus are mainly related to digital innovations - AI, machine learning, deep learning, privacy preserving technology such as blockchain technology, federated machine learning and generative adversarial network, conversational AI chatbot using natural language processing and cybersecurity (e.g., adversarial attack). At present, I am involved in 2 task forces in the International Agency for the Prevention of Blindness (IAPB) for AI in diabetic retinopathy screening, and for technology innovation for ophthalmology.

In 2019, we collaborated with the Zambia Hospital and UK colleagues to evaluate a deep learning system in detecting referable and vision-threatening diabetic retinopathy (DR)^1^. Lack of human resources, limited awareness of blindness due to DR amongst healthcare professionals, and shortage of ophthalmic services in rural areas are key challenges that need to be tackled immediately to reduce the rate of blindness in under-resourced settings. Our study demonstrates an alternative clinically effective DR screening tool using deep learning to detect referable DR, vision-threatening DR and diabetic macular edema in an under-resourced African population with diabetes, with the data prospectively collected in Zambia. Future research is beneficial to evaluate the cost-effectiveness of the AI-assisted DR screening model, aiming to develop and maintain sustainable national eye care programs to prevent DR-related blindness among the African population.

**Bram van Ginneken:** 25 years ago, I started to do a PhD. My task was simple: write a computer program that detects tuberculosis on a frontal chest radiograph. In those days the first digital x-ray machines were brought on the market and the idea was that it would be handy if the computer, where the digital images would be stored on anyway, could also make a diagnosis. It turned out to be harder than I expected. At the end of my PhD, I was much better at reading chest x-rays than my own software. And digital X-ray machines were too expensive for the under-resourced countries where TB was common. But my bold attempt was received enthusiastically, and I got the opportunity to start my own lab in medical image analysis. It took several generations of PhD students before we had well-working, CE certified software. Since that moment, in 2014, the use of the software, sold under the name CAD4TB, has rapidly increased. The software now has been shown to perform better than human readers in several large studies. Last year, the World Health Organization made a recommendation, for the first time in history, to use computer software for interpreting chest radiographs in TB screening, in place of human readers.

**Martin C. Were:** My work over the past 15 years has involved developing, implementing, and evaluating digital medicine applications for low-resource settings. Primary areas of system development and implementation focus have been in mobile health (mHealth) applications and electronic health record systems. I am Founder and Lead of the mUzima mHealth project, which is a mobile extension of the OpenMRS Electronic Medical Record System that is currently deployed in several countries. I also serve as a Health Information System Technical Advisor for the Friends in Global Health (FGH) program in Mozambique. In this role, I provide guidance on approaches for developing and implementing a range of national-level clinical information systems, with an emphasis on interoperability and compliance with national policies and international standards. My other digital medicine projects have included: developing, implementing, and evaluating computerized clinical decision support systems for HIV care and chronic disease programs in resource-limited settings; implementation science around health information systems; and developing and evaluating frameworks for equity and ethical aspects of digital health systems. I have also been involved in projects that leverage bioengineering, machine learning, and mHealth approaches for point-of-care bloodless hemoglobin level determination using inner eyelid photos and will be starting a new project to bloodlessly measure bilirubin levels through the skin. I have over 80 peer-reviewed publications across a range of digital health areas relevant to low-resource settings.

I previously served as Chief Medical Information Officer for the Academic Model Providing Access to HealthCare (AMPATH) program in Kenya, which is a large HIV care and treatment program. I continue to be involved in Health Informatics capacity building initiatives, with an emphasis on training the next generation of leaders in digital health within under-resourced settings. To this end, I founded and directed Kenya’s first Institute of Biomedical Informatics and was the lead in developing the ‘Benchmarks for the Master of Science in Health Informatics Programs in East Africa’ that will be enforced across degree programs in the region. I also previously chaired the Education Working Group of the Pan-African Health Informatics Association.

Looking at the whole healthcare pipeline, where can digital medicine help bypass limited healthcare issues and what are the priorities in the current context?

**W.H.C.:** The COVID-19 pandemic represents a unique opportunity to move forward for better collection and management of data to inform decision-making, policy development, electronic disease surveillance, monitoring, and evaluation, and highlights the need for strengthening human resource capacity for proper implementation of health information systems considering the context, needs, vulnerabilities and priorities of countries. In addition, digital medicine tools and information and communication technologies can help reduce inequalities in under-resourced settings, and those are playing an important role to promote innovation and knowledge generation in low- and middle-income countries, especially to disadvantaged groups and rural areas. Telehealth consultations have proven to be immensely helpful during the pandemic but much more needs to be done. Many of those consultations were carried out by dedicated phone lines, especially at the beginning of the COVID-19 pandemic, but due to the increasing demand for his service, many phone lines collapsed. Online consultation systems were developed and implemented for online triage of suspected COVID-19 patients, but many of those lack interoperability with electronic medical records and other health-related information systems.

**D.S.W.T.:** COVID-19 pandemic crisis has lasted for 2 years, resulting in major impacts in healthcare settings: (1) congestion and overcrowding of hospital beds with COVID-19 positive patients; (2) delay and increased in waiting time for non-COVID-19 related medical and surgical conditions; (3) increased in morbidities and mortalities of the hospital staff and patients due to COVID-19 exposure; (4) shortage of medical expertize to treat the routine medical conditions due to deployment to the frontline. Thus, this crisis has also provided huge opportunities for digital medicine in transforming deliveries of medical care. We have seen major adoptions in all range of digital technologies, including AI/machine learning/deep learning, big data analytics, internet of medical things and blockchain that can be used to perform monitoring, surveillance, detection, and prediction of COVID-19 incidence/occurrence and to mitigate and prevent the spread of COVID-19 for those disease free populations.

In the low resource settings (e.g., rural China), the adoption of blockchain has been utilized to track the supply chain in medication deliveries from pharmaceutical companies to warehouses, then to the major hospitals, rural community pharmacies and to patients’ doorsteps. At the global health landscape, we also see an enormous utilization rate for virtual medical services via tele-medicine platform to provide care for those patients from the low-resource settings by the international medical experts, and this trend will continue to grow exponentially even when COVID-19 subsides.

Nonetheless, when exporting digital tools into a new context, it is important to consider many real-world factors, including the readiness of the local infrastructures such as clinical services, equipment, treatment modalities, IT systems, tele-communication network and cost of the AI systems^2^. This will require a multi-disciplinary approach supported by the government, hospital systems, physicians, AI companies and robust digital and tele-communication systems.

**B.v.G:** Human expertize is scarce in under-resourced countries. Digital solutions should simplify clinical workflows and ideally, they should be usable after limited training. Also, costs are always important. There are now cheap portable x-ray devices, with a small battery and solar panels, that you can carry in a backpack. Add a small laptop and you can walk into any building and quickly set up a mobile clinic.

**M.C.W.:** The effectiveness and appropriateness of digital medicine in bypassing limited healthcare issues will vary greatly across different implementation settings. It is thus difficult to make blanket statements on where digital health can effectively be employed across the healthcare pipeline without taking into consideration the contextual issues surrounding each implementation. In essence, the effectiveness of any digital solution is only as good as its implementation. It should also be recognized that tools like artificial intelligence, machine learning techniques, smartphone applications, short messaging service (SMS), geo-location systems, and wearable devices are just technologies that can be applied variably across multiple use cases with varying success.

This said, frameworks exist that outline areas where digital medicine can be useful within low-resource settings. As an example, in our publication ‘*Leveraging Digital Health for Global Chronic Diseases’*^3^, we outline some categories where digital medicine can be effectively employed within low-resource settings.

When appropriately developed and implemented, the above digital health systems can positively impact patient health outcomes, strengthen health systems, overcome limitations financial and human resources, improve access to care and reduce disparities with resource-limited settings.

What are the main challenges in deploying digital medicine tools in these settings from your experience?

**W.H.C.:** There is an important need for strengthening resource capacity for the proper implementation of health information systems, considering the context, needs, vulnerabilities, and priorities of countries. In this sense, there are many technological, organizational, and socio-cultural challenges in low- and middle-income countries. These challenges include poor or inadequate infrastructure, slow or intermittent Internet connectivity, inequalities in access to electricity, adult literacy and health literacy issues, and digital health illiteracy. In addition, efficient health innovation projects and health management programs are key challenges of the health sector in resource-constrained settings. Moreover, improving health system processes to achieve health sector objectives is a relevant challenge. If processes are not mapped and improved or reviewed regularly, we might end up digitizing chaos.

The lack of interoperability between digital health solutions and mobile applications is a huge undertaking in many low- and middle-income countries; common reasons include reliance on legacy systems, lack or inconsistent use of existing standards and weak leadership and governance.

On top of that, the lack of qualified and skilled professionals in digital health is one of the main barriers for digital health application, especially in low- and middle-income countries. Many health care professionals, including physicians, are unevenly distributed and/or concentrated in urban areas, across several of those countries.

**D.S.T.W.:** Resource limitations are presently the biggest barrier to the translation of benefit from these technologies to improve public health measures, particularly in low- to middle-income countries. This takes numerous forms including lack of manpower, inadequate infrastructure, gaps in health coverage, lack of internet access, and even lack of funding for research to develop localized solutions. However, with the right configuration and localization, applications of technology can enable decentralized care and increased capacity in these settings.

Digital platforms for health data are limited by the lack of interoperability with each other, while platforms for health communication lack tools to regulate the quality of information and cybersecurity. Limitations for big data and AI include the quality of data and potential algorithmic bias. For instance, AI algorithms are often trained using data from tertiary settings rather than primary care, with poor representation of mild disease. This can have clinically significant effects such as inflated case fatality estimates. Additional limitations of AI include risks such as over-fitting to training datasets and vulnerabilities such as adversarial attacks. These can lead to unforeseen errors from incidental variations or artefacts in input data.

**B.v.G.:** Most effort goes to building tools for a Western setting, there are now 200 AI products on the market for radiology, and almost all these were launched in the last 5 years, so we see a huge increase. But most products are not relevant in under-resourced settings. We should simply develop more tools addressing the needs of clinics in countries where CT and MRI are hardly available.

**M.C.W.:** The challenges in deploying digital medicine tools in under-resourced settings will vary by country, program, and digital system. Challenges related to deploying these systems fall into several categories, with key ones being: (a) policies and strategies; (b) processes for operational standardization; (c) governance structures; (d) human capacity; (e) Infrastructure; (f) Cost; (g) Evidence for scientific validity and cost-effective benefit; and (g) long-term sustainability.

More in general, what are the main bottlenecks in a more widespread use of digital tools for healthcare in those contexts?

**W.H.C**.: Access to the Internet is still a main bottleneck in many low- and middle-income countries in part due to the lack of infrastructure deployment (especially in rural and hard-to-reach areas) and due to the cost of services. For example, Peru is the country with the highest Internet price in Latin America. While Peru has a telemedicine regulation (approved by legislative decree number 1490 in May 2020), which states that Internet and telephone providers will provide to the population with free Internet/phone access to specific telehealth services provided by the Ministry of Health, it has not been implemented yet.

Scale-up of digital health solutions may be the most promising investment in resource-constrained settings. In this sense, cost-effective digital health solutions should be scaled and sustained, so users and patients can be supported in efforts to promote health and well-being in low- and middle-income countries.

It is important to point out that there are often high-level changes and turnover in local, regional, and national governmental offices in many low- and middle-income countries, which can directly affect the deploying of digital health projects, programs, policies, and regulations; and many decision makers often deal with bureaucracy, budget restrictions, unmotivated and/or burnout workers, and staffing cuts.

In addition, partnerships between government agencies, academic institutions and organizations should be encouraged to support appropriate design, testing, and evaluation of digital health solutions and devices for usage mainly by patients and health care workers in low- and middle-income countries.

**D.S.W.T.:** The bottlenecks/challenges for AI and digital health implementation include AI performance issues, lack of generalizability, “black-box” and explainability, health economic evaluation, perception/acceptance from healthcare providers and patients, data privacy and ethical issues, cybersecurity and digital platform interoperability issues. Each of these issues often results in the “Valley of Death” during the clinical translation stage for these digital tools.

**B.v.G.:** I have been surprised how easily countries in Africa and Asia adopt computerized reading and other digital tools. The uptake of AI in my own country goes much more slowly. Doctors want to stay in control and would like to use AI only as a supportive tool. The problem with that approach is that, while it may make the doctor a little bit better and avoid an occasional mistake, it does not make doctors more productive and in the end only adds to the ever-increasing costs of healthcare. To keep healthcare affordable, you must accept full automation of certain tasks that are now performed by humans. Africa seems more open to that than Europe.

**M.C.W.:** The challenges described in the previous question variably cause bottlenecks in widespread adoption of digital tools in under-resourced settings. Key challenges to widespread use of digital tools include a combination of financing, leadership, and lack of sustainability models. In many cases, adequate funds do not exist to support large scale adoption of proven digital systems over time. Even when funds exist, they are often not optimally used, with inefficiencies evident across multiple areas. Without the appropriate leadership and organizational structures, priorities cannot be appropriately set that guide how resources should be used, what human capacity should be developed, how evidence should be vetted, and what policies should be developed, among others. Further, implementation and adoption of systems without a clear sustainability plan only leads to failure at a large scale.

Fairness in digital health applications has been advocated for ubiquitously and is a flourishing field of research. How does this connect to the challenges of deployment in under-resourced settings? How and why do these tools need to be re-tuned in those cases?

**W.H.C**.: Framing fairness in digital health applications as a purely technical problem solvable by adding more data or accurate computations is risky because it could exacerbate harms to vulnerable groups, especially in under-resourced settings. We should use a fair selection process, considering differences of race, gender, demographic disparities, disability, and other characteristics. We need to consider the complex relationships between biological, environmental, and social factors. In this sense, social determinants of health play an important role, and digital health tools need to be re-tuned accordingly. Ethical considerations are integral and essential when it comes to fairness in digital health applications.

**D.S.W.T.:** Telemedicine and AI offer an opportunity to provide a limited and valuable resource—that of the physician’s time and skillset—to a wider population in a more accessible way. It can potentially reduce health inequities, a fundamental principle of medical ethics, but there must be careful consideration of the design of services and algorithms, as well as their implementation in order to achieve this. AI democratization could be highly advantageous for under-resourced settings, using the AI developed in other countries to help overcome the manpower and expertize shortage issues to screen and diagnose complex diseases, although it is also important to have the corresponding clinical ecosystem, digital and financial structure. For the under-resourced settings, the key is to prevent life-threatening and sight-threatening conditions, and thus, the referral thresholds by these digital tools will need to be carefully set based on the health economic analysis that is feasible to the specific country.

Whilst AI might appear to be objective, biases can be inherent in the algorithms. Inherent to ML is that the algorithm learns from historic data and those under-represented in these data sets may suffer from inaccurate diagnoses, and this to a larger extent is why validation using real-world data is important. The design of these algorithms and their introduction into clinical practice should incorporate the principles of equity, so that the output does no harm. Proactive steps can be taken at each step of the data collection, training and evaluation stages, such as broad stakeholder engagement, ensuring data represents the protected groups and that such data is identifiable to guard against cohort bias, and formulating systems to continually evaluate key metrics across different groups. Thus, it is crucial to take into account the clinical, digital, and health economic consideration for AI adoption especially in the low-to-middle income setting countries.

**B.v.G.:** I’ve seen this question more often and the underlying assumption is that tools developed in high-resource countries will be used as-is in low-resource settings. That is highly unlikely to work. Any AI product typically needs to be tailored to the target population. We’ve trained our software for TB detection always using data from TB screening programs in Africa, Asia and elsewhere. Having a diverse training set and performing validation studies on data from different settings is essential. You can interpret AI fairness in many ways, but typically you just need to evaluate performance on different groups, male versus female, young versus old, different ethnicities, people with underlying disease, prior TB, etc.

**M.C.W:** Individuals, organizations, and countries in resource-limited settings need the same protections as those in higher-resource settings when it comes to adoption and deployment of digital health systems, and in use of information and data derived from these systems. As such, double standards should be avoided at all costs, even in settings that lack guidance for systems deployment, security, and data use. Fairness in digital health systems therefore requires transparency on the technologies and algorithms used in digital health tools—the systems should not be ‘black boxes’ which implementers cannot understand or decipher. The systems and algorithms also must be adequately tested within the implementation context with assurance that outputs are as expected. From the outset and at all stages of algorithm development, potential biases need to be addressed, with assurance that all groups benefit equally and that no group is systematically disadvantaged by the system. If inputs in development or testing come from under-resourced settings, technology transfer mechanisms should be employed to ensure fair access and benefits to the derived intellectual property.

Fairness in deployment requires implementation of digital systems that reduce social inequality for the whole population, without exacerbating the digital or health divide. As such, adopted systems should take into consideration challenges in existing infrastructure, cost, human capacity and inability of select groups to effectively use the digital tools. Where risk exists that would discriminate against individuals or groups, appropriate mitigation measures should be employed. Further, the decision around deploying a solution should be gauged against their cost-benefit relative to other proven medical interventions. In some cases, it would not be advisable to divert limited resources from more proven interventions, unless the collective cost-benefit ratio of the digital solution is determined to be higher.

What are your point of view and experience on the way these projects are carried out from the perspective of transfer of knowledge and competence?

**W.H.C**.: It is necessary to move from digital health pilot projects to scalable and sustainable solutions. Many projects do not scale due to organizational, physical, political, technological, and socio-technical challenges. In this sense, it is important not only the funding support of governmental agencies for digital health projects and programs but also the development of international partnerships between institutions promoting North-to-South, South-to-North, and South-to-South collaborations. Strengthening existing networks on digital health of public and private institutions with academic centers who have experience should be supported.

Digital health projects need to incorporate the local context, consider the local needs, and be sensitive to the local economic, social, cultural, and organizational factors. Innovative intercultural and multilingual digital health tools are needed, especially in underserved areas. The ethical challenges, socio-technical and cultural issues, including resistance to change need to be addressed in digital health projects and programs. Financial incentives and strong political commitment and leadership are important factors to consider.

Finally, there is an urgent need to develop educational programs on digital health^4^ at different levels (decision-makers and managers, digital health implementers and for health professionals); and we need more culturally adapted educational digital health programs for patients and users.

**D.S.W.T.:** To effectively implement AI and digital solutions in real-world settings, education and knowledge transfer is key. It is always important for the hospital providers to understand the principles of AI and digital solutions applications within the healthcare settings. To improve the AI and digital literacy in the clinical settings, the university and hospital senior leadership could look into starting some of the basic curriculums within the undergraduate or residency training period, or sending the fellows or faculties overseas for sabbaticals to acquire AI and digital skills. The principle of AI application can also be summarized into 5 rights: (1) Right intended use environment; (2) Right clinical dataset; (3) Right technical methodology; (4) Right AI architecture; (5) Right AI implementation (safety and cost-effectiveness). These 5 considerations are crucial in defining the success of AI adoption within the real-world clinical settings.

**B.v.G.:** Over the years, I have had the pleasure to work with many groups in Africa and Asia. You can only develop medical software if you work with physicians who use the product, provide feedback and validate that it works according to the intended use. In a new project, we develop a solution for ultrasound screening of pregnant women, using a low-cost probe attached to a smartphone. The real-time deep learning analysis is running on the phone, guides the midwife who acquires the data and detects a high-risk pregnancy. We work with midwives in four African countries. Developing AI software using deep learning has become a much easier process with so much open-source software and free training resources available. I expect that in the future more products are developed and maintained in Africa.

**M.C.W.:** Knowledge transfer for digital health projects often needs to occur for various categories of personnel, among them being developers, system implementation teams, end users, leadership, and consumers of data outputs. As such, effective knowledge transfer requires a clear understanding of both the capabilities of targeted groups as well as the context within which the system is being deployed. Oftentimes, multi-modal mechanisms for knowledge transfer need to be adopted. A core knowledge transfer modality is adequate codification of materials through easily accessible documentation and other information, education, and communication materials. Historically, in-person workshops and hands-on training at implementation sites have played a significant role in personalizing knowledge transfer. However, in the age of the COVID-19 pandemic that has limited in-person gatherings and travel, recorded content and webinars are increasingly being used.

For developers, hackathons and bootcamps are sometimes used to provide learning ecosystems for imparting needed competencies. Some digital health projects have deployed demonstration instances of their solutions for a realistic experience of the solution in action. For larger projects, formal and informal communities organized around various categories of personnel (implementer, developer, and user communities) or even by country and region have emerged. More mature systems, such as OpenMRS and DHIS, have developed certification programs and even ‘universities’ that aim to impart key competencies with clear recognition of achievements by learners.

The effectiveness of knowledge transfer mechanisms can vary widely within under-resourced settings. It is evident that knowledge transfer requires deliberate resource planning that involves personnel time and financial costs. It is not uncommon to find projects where comprehensive resource allocation to support knowledge transfer is not included in contracts. Smaller digital health projects might not even have the team that can develop high-quality knowledge transfer materials, or that can train all end-user groups– as an example, these teams might have very little experience and knowledge of the implementation setting to effectively support actual implementations. Perceived competition and the need to secure intellectual property can sometimes also limit how freely knowledge is shared. For this reason, open-source systems are often strongly advocated in under-resourced settings, even though questions remain on how effectively open-source approaches can be implemented without disincentivizing innovators. In some cases, such as those involving Artificial Intelligence and Machine learning algorithms, details of the system can be opaque to users, and approaches are needed to address this gap.

*This interview was conducted by Lorenzo Righetto*.
